# Optimising Immunogenicity with Viral Vectors: Mixing MVA and HAdV-5 Expressing the Mycobacterial Antigen Ag85A in a Single Injection

**DOI:** 10.1371/journal.pone.0050447

**Published:** 2012-12-21

**Authors:** Gareth Betts, Hazel Poyntz, Elena Stylianou, Arturo Reyes-Sandoval, Matthew Cottingham, Adrian Hill, Helen McShane

**Affiliations:** 1 Nuffield Department of Surgery, Oxford University, John Radcliffe Hospital, Oxford, United Kingdom; 2 The Jenner Institute, Oxford University, Oxford, United Kingdom; Instituto Butantan, Brazil

## Abstract

The Bacillus Calmette - Guerin (BCG) vaccine provides a critical but limited defense against *Mycobacterium tuberculosis (M.tb)*. More than 60 years after the widespread introduction of BCG, there is an urgent need for a better vaccine. A large body of pre-clinical research continues to support ongoing clinical trials to assess whether viral vectors expressing *M.tb* antigens that are shared by BCG and *M.tb*, can be used alongside BCG to enhance protection. A major focus involves using multiple unique viral vectors to limit anti-vector immunity and thereby enhance responses to the insert antigen delivered. The successful introduction of viral vector vaccines to target *M.tb* and other pathogens will be reliant on reducing the costs when using multiple vectors and inhibiting the development of unwanted anti-vector responses that interfere with the response to insert antigen. This study examines methods to reduce the logistical costs of vaccination by mixing different viral vectors that share the same insert antigen in one vaccine; and whether combining different viral vectors reduces anti-vector immunity to improve immunogenicity to the insert antigen. Here we show that a homologous prime-boost regimen with a mixture of MVA (Modified Vaccinia virus Ankara) and Ad5 (human adenovirus type 5) vectors both expressing Ag85A in a single vaccine preparation is able to reduce anti-vector immunity, compared with a homologous prime-boost regimen with either vector alone. However, the level of immunogenicity induced by the homologous mixture remained comparable to that induced with single viral vectors and was less immunogenic than a heterologous Ad5 prime-MVA-boost regimen. These findings advance the understanding of how anti-vector immunity maybe reduced in viral vector vaccination regimens. Furthermore, an insight is provided to the impact on vaccine immunogenicity from altering vaccination methods to reduce the logistical demands of using separate vaccine preparations in the field.

## Introduction

Disease caused by *Mycobacterium tuberculosis* (*M.tb*) causes high levels of morbidity and mortality, accounting for approximately 1.5 million deaths worldwide annually [1]. The Bacillus Calmette - Guerin vaccine (BCG) has been in widespread use for over 60 years and is the only currently licensed *M.tb* vaccine. Although BCG is largely ineffective at protecting against adult pulmonary disease, it does confer significant protection against disseminated disease in infants [2] and it is likely that either the current or a modified BCG strain will continue to be employed in the foreseeable future. A number of subunit vaccines designed to boost BCG are currently in clinical trials [3], [4], including candidates that contain antigens shared by BCG and *M.tb*.

*M.tb* vaccine development is hampered by a lack of biomarkers to predict efficacy. However, observations in immunocompromised mouse models and patients have identified factors associated with an elevated risk of disease that are essential but not sufficient for protection. These include CD4+ T cells, as highlighted in HIV infected subjects [5] and in mice with an underlying genetic deficiency of the MHCII processing pathway [6]; and CD8+ cells [7]. Mice and patients with a genetic deficiency of the IFN-γ pathway are susceptible to mycobacterial infection [8], [9] and patients that have received monoclonal anti-TNF-α blocking antibody therapy are more susceptible to reactivation of latent *M.tb* [10]. Research has shown that CD4+ cells that co-produce TNF-α, IL-2 and IFN-γ cytokines (3+), termed polyfunctional cells, have been associated with protection against *Leishmania* in mice and are present following BCG vaccination of mice [11] and after boosting with a candidate *M.tb* vaccine MVA85A in humans [12].

Attenuated viral vectors engineered to express subunit antigens generate high levels of cellular immunity. MVA expressing the mycobacterial antigen 85A (MVA85A) is currently being evaluated in clinical trials [13] and has been shown to boost Ag85A specific CD4+ responses following a BCG prime [14]. Efficacy against *M.tb* challenge has been demonstrated in several animal models including a veterinary target species (cattle) [15], [16]. Importantly, MVA85A has also been shown to induce high levels of antigen 85A (Ag85A) specific CD4+ cells in BCG vaccinated subjects [12], [17]. Recombinant adenoviral vectors were shown to be effective at inducing antigen specific CD8+ cells in clinical trials of HIV [18] and malaria vaccines [19]. Ad5 expressing Ag85A induced high levels of Ag85A specific CD8+ cells and conferred protection from *M.tb* in mice [20], [21]. A clinical trial in South African adults evaluating a recombinant human adenovirus type-35 expressing the *M.tb* antigens Ag85A, Ag85B and TB10.4 (AERAS-402) demonstrated high levels of antigen specific CD8+ T cells [22]. Although these studies are encouraging, multiple administrations with subunit vaccines may be required. The development of anti-vector immunity to viral vectors after administration limits repeated use of the same viral vector to boost immunity. Sequentially administering two different viral vectors expressing the same antigen, known as a heterologous prime boost regimen, elicits high levels of cellular immunity [23]–[25]. This approach ensures anti-vector immunity recognising the first virally vectored vaccine does not attenuate the recognition of the shared insert antigen expressed by the viral vectored vaccine in the boost. A vaccine regimen using both an adenoviral and MVA vaccine to boost sequentially after BCG vaccination maybe necessary to optimse immunogenicity and therefore enhance efficacy, but delivery of this regimen may be logistically challenging. The evaluation of ways to reduce the logistical costs of a *M.tb* vaccine regimen that could include up to three separate vaccinations is required. We investigated whether multiple doses of a mixture of MVA and adenoviral vectors expressing Ag85A insert antigen in a single vaccine could induce immunity similar to that observed in a heterologous regimen using sequential doses of different viral vectors. We hypothesised that mixing different vial vectors in a single vaccine would reduce anti-vector immunity against each vector and induce a response to Ag85A insert antigen comparable to that seen with sequential vaccination with different viral vectors expressing Ag85A. In mice, responses to the insert antigen in MVA and adenoviral viral vectors are known to peak at approximately 1 [26] and 2–3 weeks [25], [26] respectively. We compared levels of immunity to the insert antigen and viral vector backbone using this mixing strategy with both homologous and heterologous prime-boost regimens.

## Methods and Reagents

### Vaccinations

All experiments were performed with 6- to 8-wk-old female BALB/c mice (Harlan Orlac). All procedures were carried out under the terms of the UK Animals (Scientific Procedures) Act Home Office Project Licence (UK Home Office PPL 30/2412) and were approved by the University of Oxford Animal Care and Ethical Review Committee.

Mice were either vaccinated intradermally (i.d.) in each ear (25 µl/ear) or intranasally (i.n.) (50 µl). The same doses of vaccine were given via either route of vaccination. Groups of 5–6 mice per group were vaccinated with either of the following vaccine preparations: 5×10^6^ plaque forming units (PFU) of MVA85A (MVA), 5×10^9^ virus particles (vp) of HAdV-5.85A (Ad5), or both 5×10^6^ PFU MVA85A and 5×10^9^ vp HAdV-5.85A combined in the same volume of vaccine (Ad5+M). In one experiment, mice were injected i.d. with approximately 1.5×10^5^ CFU SSI BCG (Statens Serum Institute).

### Ex-vivo IFN-γ ELISpot assay

Ammonium chloride lysis buffer (ACK)-treated peripheral blood mononuclear cells (PBMC) or splenocytes were stimulated with 10 µg/ml of purified protein derivative-tuberculin (PPD-T) (Statens Serum Institute), 2 µg/ml of the Ag85A immunodominant CD4+ T cell specific P15 (TFLTSELPGWLQANRHVKPT) peptide and CD8+ T cell specific P11 (EWYDQSGLSVVMPVGGQSSF) peptide in separate IPVH coated wells for 18 hours, as described previously [27], [28]. A titration of cells per well was run throughout all experiments to ensure the frequency of spot forming units (SFU) per well could be accurately counted, beginning with 2.5×10^5^ cells per well for splenocytes or 50 µl of blood for PBMC. The number of PBMC per well was calculated retrospectively. In some experiments anti-vector immunity was measured by stimulating cells with peptides comprising documented Ad5 H-2^d^ restricted T cell epitopes including Ala (LPKLTPFALA) and KiA (KYSPSNVKIA) [29] and MVA epitopes including F2G (SPGAAGYDL), E3 (VGPSNSPTF) [30] and C6(S) [31]. The vaccinia virus sequence, GFIRSLQTI, is different to the orthologous sequence in MVA, SFIRSLQTI, so in the MVA C6(S) peptide, the G was mutated to S. Documented KiA and Ala T cell epitopes were manufactured with the addition of a terminal alanine residue and therefore are referred to by these names throughout, whereby the documented peptides hex3 (KYSPSNVKI) and dbp7 (LPKLTPFAL) with an additional alanine residue in our hands are referred to as KiA and Ala respectively. 2.5×10^5^ naïve splenocytes were cultured together with PBMC to present peptides.

### Flow cytometry

Cells were harvested from spleen, bronchoalveolar lavage (BAL), mediastinal lymph nodes (LN) and lungs. BAL samples were collected by three successive lavages of each lung with 0.5 ml 10 mM EDTA PBS (Sigma). Lungs were perfused with PBS and cut into small pieces before digesting with DNase and collagenase (Sigma). Cells were stimulated in separated wells with pooled 85A peptides and the vector T cell epitopes listed above for 2 hrs at 37°C. GolgiPlug (BD Biosciences) was then added and cells were incubated for a further 4 hrs before overnight incubation at 4°C. Intracellular cytokine staining was performed the following day. Cells were washed and stained with violet LIVE/DEAD dead cell exclusion marker (Invitrogen) according to the manufacturer's instructions and anti-CD4, CD8, CD3, CD45R/B220 (eBioscience). CD45/B220+ cells were excluded from analysis. Intracellular cytokine staining (ICS) was performed with anti-IL-2, TNF-α and IFN-γ antibodies (eBioscience) using the BD Cytofix/Cytoperm kit according to the manufacturer's instructions. Samples were run on a LSRII flow cytometer (BD) and analysed using FlowJo software, before analysis and presentation of distributions using SPICE version 5.1, downloaded from <http://exon.niaid.nih.gov/spice> [32].

### Statistical analysis and graph generation software

Prism 5 graph pad software was used to generate graphs and perform statistical analysis. Comparisons between vaccine regimens were performed using two-tailed Mann-Whitney test to compare groups of mice at the same time point that received different vaccines and two-tailed Wilcoxon matched-pairs signed rank test were performed to compare between the same mice at different time points after vaccination.

## Results

### Comparison of Ag85A-specific immunity generated by a homologous prime-boost regimen using either a mixture of Ad5 and MVA viral vectors expressing Ag85A in the same vaccine preparation with a homologous prime-boost regimen using either Ad5 or MVA alone

**Figure 1:**
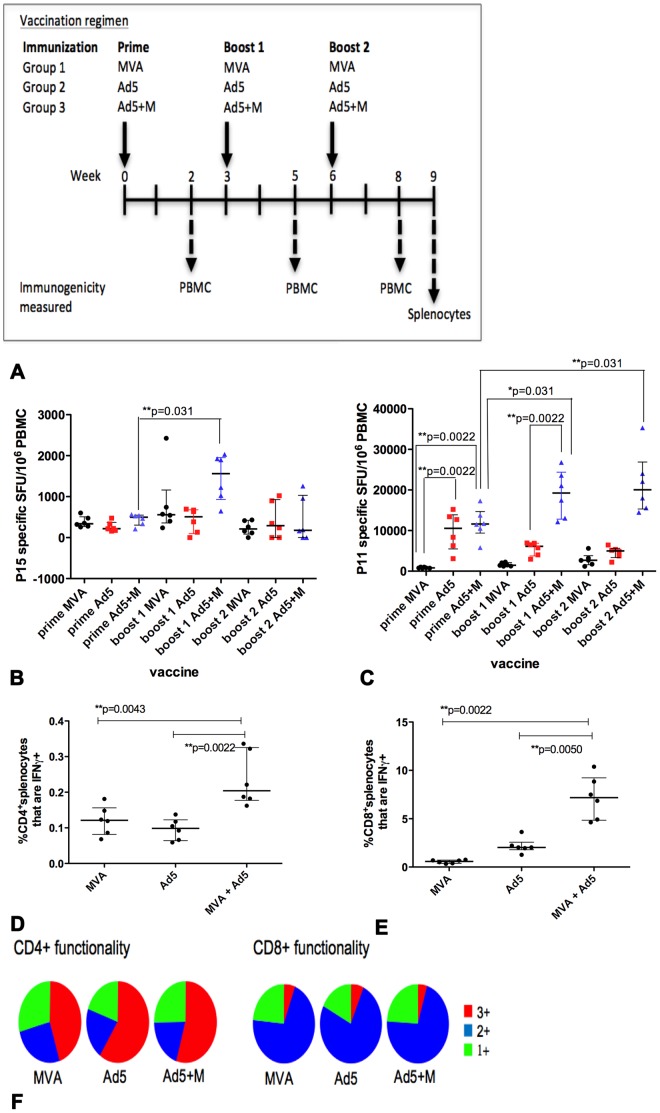
Measurement of vaccine induced immunogenicity two weeks after each vaccination. Groups of mice (n = 6/group) were primed i.d. with Ad5, MVA or Ad5+M expressing Ag85A and received a homologous boost at 3 weeks and 6 weeks after the prime (A). IFN-γ release by PBMC stimulated with the BALB/c Ag85A immunodominant CD4+ T cell epitope p15 (B) and CD8+ T cell epitope p11 (C) was measured 2 weeks after each vaccination by ELISpot. Splenocytes were stimulated with the total Ag85A peptide pool 3 weeks after the final vaccination and IFN-γ, IL-2 and TNF-α intracellular cytokine staining was measured by flow cytometry. The frequency of CD4+ IFN-γ+ (D) and CD8+IFN-γ+ splenocytes (E) and the number of cytokines co-produced (1+, 2+, 3+) in CD4+ and CD8+ splenocytes (F) was analysed. 1+ and 2+ indicates production of any one or two of the three cytokines measured respectively. 3+ indicates co-production of all three cytokines. Data is representative on one experiment. The median and interquartile range is displayed (B–E).

Throughout this study, immunogenicity was measured in PBMC to enable the effect of sequential vaccinations to be measured more accurately in the same group of mice, as well as in splenocytes after the final vaccination. For initial experiments, groups of mice received three injections of viral vaccines encoding Ag85A as part of a homologous prime-boost-boost vaccination schedule (Figure 1a). Group 1 received MVA; group 2 received Ad5; and group 3 received Ad5+M. Mice were boosted with the same vaccine at three and six weeks after the prime. Immunogenicity in PBMC was measured 2 weeks after each vaccination and in splenocytes 3 weeks after the final vaccination. CD4+ PBMC Ag85A-specific T cell responses were equivalent in all groups after the prime (Figure 1b). CD8+ PBMC Ag85A-specific T cell responses were similar in mice that received Ad5 or Ad5+M and significantly higher than those primed with MVA (Ad5, **p = 0.0022; Ad5+M, **p = 0.0022), reflecting the established finding that adenovirus is a more potent vector for induction of CD8+ T cell specific responses than MVA [24] (Figure 1c).

Ag85A specific immunity in PBMC was not boosted in mice that received a second homologous vaccination of either Ad5 or MVA compared to the response after the prime. However CD4+ (**p = 0.031) and CD8+ T cell (*p = 0.031) Ag85A-specific T cell responses were significantly increased in mice that received a second vaccination of Ad5+M compared to the response after the prime (Figure 1b, c respectively). A second boost with the Ad5 + M mixture failed to enhance Ag85A responses further. CD4+ T cell responses in PBMC declined to a similar level as was observed in mice injected three times with a single vector (Figure 1b). Ag85A-specific CD4+ T cell splenocyte responses remained elevated in the Ad5+MVA group compared to the Ad5 (**p = 0.0022) and MVA (**p = 0.0043) group (Figure 1d), indicating these cells migrated from the blood to the spleen. This could explain why these cells did not remain at a higher frequency in blood. CD8+ T cell responses were the highest in the Ad5+M group in both the blood and spleen after three injections (Figure 1c, e respectively).

The functional profile of Ag85A-specific cells was assessed by measuring co-production of IFN-γ, TNF-α and IL-2. Antigen-specific CD4+ and CD8+ T cell splenocytes in all groups had a similar polyfunctional profile (Figure 1f) and the increased frequency of Ag85A specific cells in mice vaccinated with Ad5+M was not associated with any change in functionality.

**Figure 2:**
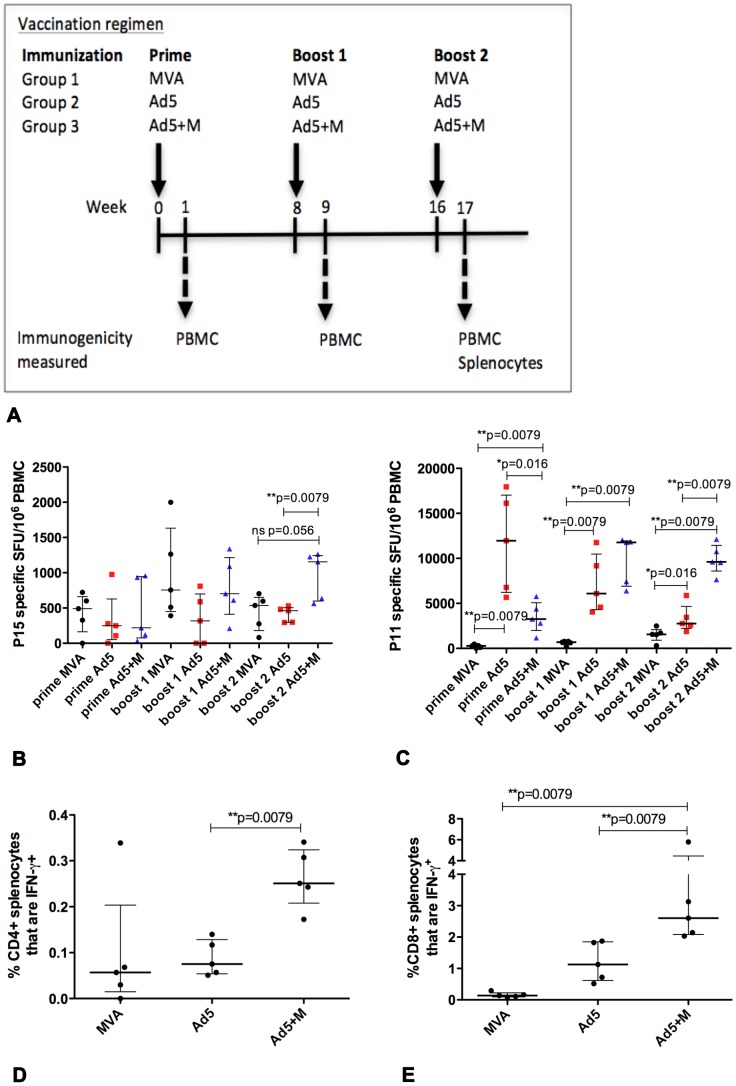
Measurement of vaccine induced immunogenicity one week after each vaccination. Groups of mice (n = 6/group) were primed i.d. with Ad5, MVA or Ad5+M expressing Ag85A and received a homologous boost at 8 weeks and 16 weeks after the prime (A). IFN-γ release by PBMC stimulated with the CD4+ epitope p15 (B) and CD8+ epitope p11 (C) was measured 1 week after each vaccination by ELISpot. The frequency of CD4+ IFN-γ+ (D) and CD8+IFN-γ+ splenocytes (E) 1 week after the second boost was evaluated by intracellular staining. Data is representative on one experiment. The median and interquartile range is displayed (B–E).

The vaccination schedule was modified to investigate whether the enhancement of CD4+ T cell responses in the Ad5+M group compared to mice vaccinated with single vectors could be increased further. The time interval between the first and second boost was extended from 3 to 8 weeks whilst the 3 weeks interval between the prime and first boost was maintained. Antigen specific PBMC CD8+ T cell responses from mice vaccinated with Ad5+M were present at a significantly higher frequency than those in mice that received Ad5 alone after both first and second boost (*p = 0.041, *p = 0.026 respectively; data not shown). A trend of enhanced CD8+ T cell responses in the spleen in Ad5+M compared to Ad5 alone vaccinated mice was also evident (p = 0.065; data not shown). In this experiment, PBMC CD4+ T cell responses were not enhanced in the Ad5+M group compared to mice injected with Ad5 or MVA alone (data not shown); or compared to the Ad5 group in the spleen. Splenic CD4+ T cell responses were higher in the Ad5+M group compared to the MVA group (*p = 0.013, data not shown). These data suggested that, in contrast to initial observations, multiple doses of mixture of two viral vectors do not consistently induce greater levels of Ag85A-specific immunity compared to multiple doses of a single vector. To further explore this possibility, we examined whether an enhancement to Ag85A immunity by using a mixture of two viral vectors instead of single vectors in a homologous prime-boost required a greater interval between the prime and first boost. The interval between the prime and first boost was therefore extended from 3 to 8 weeks, and the interval between the first and second boost was maintained at 8 weeks (Figure 2a). As MVA viral vectors are capable of eliciting strong CD4+ T cell responses and are known to peak at one week after vaccination, the decision was taken to shorten the time point to observe immunogenicity from 2 weeks to 1 week post vaccination.

Mice that received three vaccinations with Ad5+M had a higher frequency of CD4+ T cell responses in PBMC (**p = 0.0079) and splenocytes (**p = 0.0079) compared to mice vaccinated with Ad5 (Figure 2b, d); however, the Ad5+M failed to generate a higher frequency of CD4+ T cell responses compared to mice vaccination with MVA (Figure 2b, d). CD8+ T cell responses to Ag85A were increased in PBMC and splenocytes in mice that received Ad5+M compared to mice vaccinated with either MVA (**p = 0.0079, **p = 0.0079) or Ad5 (**p = 0.0079, **p = 0.0079) (Figure 2c, 2e respectively). A combination of two viral vectors in the Ad5+M group may require more time to achieve the optimal presentation of antigen to elicit maximum immunity. It is possible that reducing the time point to observe immunogenicity to 1 week post prime (Figure 2c) from 2 weeks post prime (Figure 1c) accounts for the reduced size of the CD8+ IFN-γ+ PBMC response in the Ad5+M group following the prime vaccination.

Similar experiments were performed in parallel to deliver multiple doses of Ad5+M, Ad5 or MVA into the airway by intranasal vaccination (*i.n.*). Analysis of lung parenchyma and broncho-alveolar lavage (BAL) demonstrated a pattern for the Ad5+M to generate a greater level Ag85A-specific immunity. However, in a similar manner to that observed after *i.d.* vaccination, this enhancement was not consistent. CD4+ and CD8+ T cell Ag85A-specific responses in lung parenchyma were greater in mice receiving three doses of Ad5+M group and within the CD4+ T cell compartment in BAL compared with multiple doses of Ad5 or MVA single viral vectors (data not shown). However Ag85A specific CD8+T cell responses in BAL were not consistently greater in mice receiving three doses of Ad5+M compared to three doses of Ad5 (data not shown).

### Combining a BCG prime with successive Ad5+M boosts

**Figure 3:**
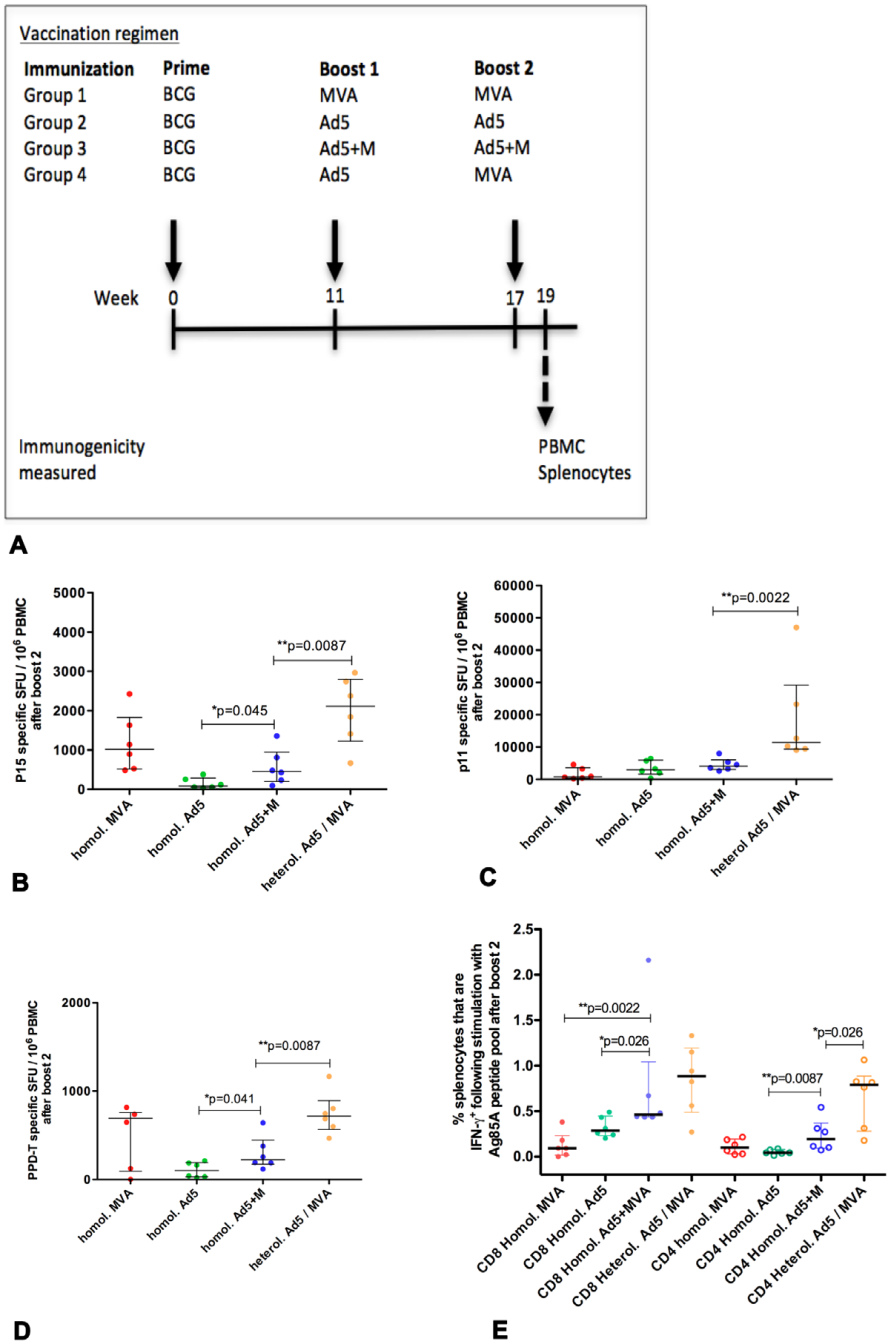
Measurement of vaccine induced immunity following a BCG prime and boosting. Mice were primed i.d. with BCG and boosted i.d. at 11 and 17 weeks after the prime with successive homologous (homol.) injections of Ad5, MVA, Ad5+M mixture or a heterologous (heterol.) vaccination with Ad5 followed by MVA (A). Antigen specific IFN-γ release was measured by ELISpot from PBMC isolated from tail bleeds 2 weeks after the second boost (B–D). PBMC were stimulated with P15 (B), P11 (C) or PPD-T (D). Splenocytes were harvested 2 weeks after the second boost and stimulated with Ag85A peptide pool to measure Ag85A specific intracellular IFN-γ production by CD8+ (filled symbols) and CD4+ (empty symbols) cells (E). Data is representative on one experiment. The median and interquartile range is displayed (B–E).

It is likely that a successful new *M.tb* vaccine regimen used in the field will incorporate BCG. Given the trend for a homologous prime-boost regimen using Ad5+M to generate greater Ag85A-specific immunity than when using MVA or Ad5 alone, further experiments were conducted to establish whether Ag85A immunity became consistently greater using two boosts of Ad5+M following a BCG prime (Figure 3a). Levels of Ag85A immunity was compared to that generated with two homologous boosts with MVA or Ad5 after a BCG prime.

I.d. BCG vaccination induced approximately 200 PPD-T specific SFU/10^6^ PBMC (data not shown) and low responses to the immunodominant Ag85A peptides P11 and P15. (3/4 mice elicited peptide P11 specific CD8+ T cells and 1/4 mice elicited peptide P15 specific CD4+ cells with an average IFN-γ elispot response of 70 SFU and 3 SFU/1×10^6^ splenocytes respectively; data not shown) when analysed the day before the first boost.

Mice were subsequently boosted twice i.d. with viral vectors. In BCG primed mice, PBMC CD4+ and CD8+ T cell responses to Ag85A were not consistently greater in mice boosted twice with Ad5+M compared to those receiving two boosts of either MVA or Ad5 alone (Figure 3b–d). Splenic CD8+ T cell responses were significantly enhanced in the Ad5+M group compared to either MVA or Ad5 single vectors (Figure 3e; **p = 0.0022, *p = 0.026 respectively). Splenic CD4+ T cell Ag85A responses were greater following two homologous Ad5+M boosts following a BCG prime compared to that with Ad5 (Figure 3e; **p = 0.0087) but not MVA (Figure 3e). Overall, two homologous boosts with Ad5+M after a BCG prime did not consistently boost immunity beyond that observed using a regimen of two injections of single vectors after a BCG prime.

### Comparison of immunogenicity generated by heterologous Ad5 prime and MVA boost with a homologous Ad5+M prime boost regimen

Given the trend that the homologous Ad5+M prime-boost regimen is able to generate stronger levels of Ag85A-specific immunity compared with two vaccinations of Ad5 or MVA alone, we wanted to determine the level of Ag85A immunity in comparison to a heterologous prime-boost regimen using an Ad5 followed by MVA.

**Figure 4:**
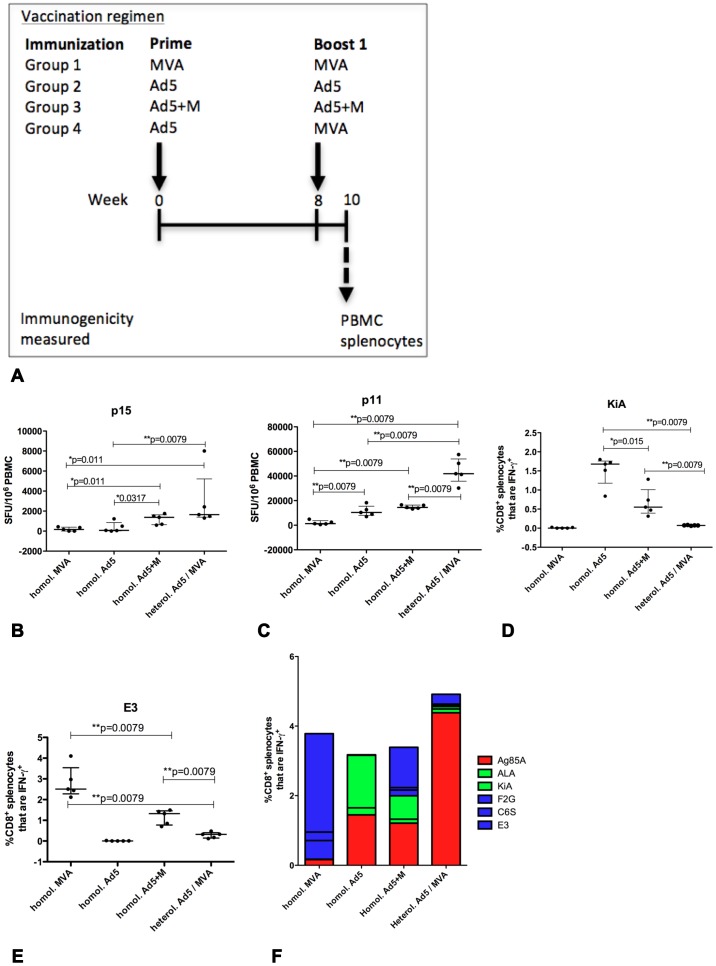
Measurement of anti-vector immunity following vaccination. Groups of (n = 5) mice were primed i.d. with Ad5, MVA or Ad5+M expressing Ag85A and received a homologous boost 8 weeks later (A). An additional group of mice (n = 6) received a heterologous prime-boost with an Ad5 prime followed by an MVA boost 8 weeks later. PBMC (B–C) and splenocytes (D–F) were harvested from all groups two weeks after the boost. PBMC were stimulated with the immunodominant CD4+ epitope P15 and CD8+ T cell epitope P11 (B, C respectively) to measure IFN-γ release by ELISpot. Splenocytes were stimulated with KiA and E3 anti-vector T cell epitopes (D, E respectively) before ICS and gating on CD8+ splenocytes. The median and interquartile range is displayed (B–E). The average frequency of CD8+ splenocyte IFN-γ+ responses to P11 and ALA, KiA, F2G, C6S and E3 vector specific epitopes by mice in each vaccine regimen, as measured by ICS, is displayed (F). Data is representative on one experiment.

BCG primed (Figure 3a) or naive (Figure 4a) mice received two injections with viral vectored vaccines. Mice received two vaccinations of either MVA, Ad5, Ad5+M or Ad5 followed by MVA. Without prior priming with BCG, an enhancement of Ag85A specific immunity by administering a homologous Ad5+M prime-boost compared to a homologous prime-boost with Ad5 or MVA was not evident amongst CD8+ or CD4+ splenocytes (data not shown) or in CD8+ T cells in blood (Figure 4c) compared to two injections of Ad5. However Ag85A responses within CD4+ T cells in blood were significantly more frequent following the homologous prime-boost with Ad5+M compared to two injections of MVA or Ad5 (Figure 4b; *p = 0.011, *p = 0.0317 respectively). The capacity of a heterologous prime-boost regimen to generate Ag85A specific immunity in mice without a BCG prime was evaluated. The frequency of both CD4+ and CD8+ T cell Ag85A responses were significantly greater in the spleen following a heterologous prime-boost compared to a homologous prime-boost with two vaccinations of either Ad5+M (**p = 0.0079; **p = 0.0079 respectively), Ad5 (**p = 0.0079; **p = 0.0079 respectively), or MVA (**p = 0.0079; **p = 0.0079 respectively) (data not shown). Ag85A specific immunity was also greater in CD8+ T cell PBMC (Figure 4c) in mice that received the heterologous prime-boost compared to injection of either Ad5 (**p = 0.0079); MVA (**p = 0.0079); or Ad5+M (**p = 0.0079). CD4+ T cell responses in blood were greater in mice that received the heterologous prime-boost than either Ad5 (**p = 0.0079) or MVA (*p = 0.011) homologous prime-boost (Figure 4b) but, unlike in the spleen, were not greater than those vaccinated with homologous Ad5+M prime-boost (Figure 4b).

Overall, it was apparent that a a hierarchy of Ag85A immunogenicity was generated by the differnent vaccine regimens using multiple injections of viral vectors without a BCG prime. There was a trend for an increasing level of responses observed in mice that received the homologous A+M regimen compared to those vaccinated with a homologous regimen using a single vector. The highest levels of immunity was observed in mice that received the heterologous prime-boost regimen.

A similar finding was observed following a BCG prime. Ag85A responses were greater after heterologous Ad5 – MVA boosts, compared with two homologous boosts with Ad5+M. CD4+ T cell Ag85A responses were consistently higher in PBMC (**p = 0.0087, Figure 3b) and splenocytes (Figure 3e, *p = 0.026) after the final injection. CD8+ T cell Ag85A responses in PBMC after the final injection were also greater in mice that received a heterologous boost with Ad5 and MVA successively compared to two homologous boosts with Ad5+M (**p = 0.0022; Figure 3c). A similar finding was also observed in response to PPD-T by PBMC (Figure 3d).

### Comparison of anti-vector immunity elicited during each vaccine schedule

Analysis of T cell mediated anti-vector responses provides an insight to the extent that antigen presentation of insert antigen and persistence of viral vector is inhibited by T cell immunity as well as the proportion of the overall T cell response that is detracted from recognising Ag85A insert antigen. We therefore evaluated CD8+ T cell mediated anti-vector immunity in all three vaccination regimens two weeks after the boost in mice that were not primed with BCG (Figure 4). Predefined, well characterised CD8+ T cell peptide specific responses to the MVA and Ad5 vectors were analysed following vaccination. Responses to Ad5 T cell epitope KiA were lower in splenocytes of mice that received two injections of Ad5+M compared to those vaccinated with two injections of Ad5 vector alone (*p = 0.015, Figure 4d). Responses to MVA vector T cell epitopes C6S, F2G (*p = 0.031, *p = 0.015 respectively, data not shown) and E3 (**p = 0.0079; Figure 4e) were lower in splenocytes of mice that received Ad+M compared to those vaccinated with MVA. There was also a lower frequency of anti-MVA specific responses to C6S (*p = 0.015) and F2G (*p = 0.0079) responses present in PBMC of mice that received Ad5+M after boosting compared to those vaccinated with MVA (data not shown). Although the mixing of vectors did reduce anti-vector immunity compared with homologous boosting using single vectors, the magnitude of both Ad5 specific CD8+ KiA (spleen, **p = 0.0079, Figure 4d) and Ala peptides (PBMC, **p = 0.0079, data not shown) and MVA vector specific responses to CD8+ E3 (spleen **p = 0.0079, Figure 4e), F2G (spleen, **p = 0.0079; PBMC **p = 0.0079; data not shown) C6S peptides (PBMC, *p = 0.011, data not shown) were significantly greater in mice that received a homologous prime-boost with Ad5+M than in mice that received the heterologous prime-boost regime.

The total Ad5 CD8+ KiA and ALA specific peptide splenocyte responses of each mouse as determined by flow cytometry were summed and, separately, total CD8+ MVA specific E3, F2G and C6S peptide responses were summed (data not shown). The summed anti-Ad5 (**p = 0.0079) and anti-MVA (**p = 0.0079) vector peptide specific response were significantly reduced following a heterologous prime-boost regimen compared to the homologous A+M prime-boost (data not shown). In turn, the summed anti-Ad5 (*p = 0.0159) and anti-MVA (**p = 0.0079) vector peptide specific responses were significantly reduced following the two vaccinations with the Ad5+M mixture compared to the homologous prime-boost with two vaccinations of Ad5 or MVA respectively (data not shown). Summed anti-Ad5 and MVA vector peptide specific immunity were also significantly reduced in the heterologous prime-boost regimen compared to the homologous prime-boost regimen using two vaccinations of Ad5 (p = 0.0079) or MVA vector (**p = 0.0079) respectively (data not shown).

The average CD8+ IFN-γ+ T cell peptide response measured using ICS to each vaccine regimen against Ag85A and each anti-vector peptide response was calculated and compiled to show the frequency of the total observed CD8+ IFN-γ+ T cell peptide response and the breakdown of each response to the total CD8+ IFN-γ+ T cell peptide response by each regimen (Figure 4f). The combined average responses to Ala and KiA Ad5 vector T cell specific peptides and combined average responses to F2G, C6S and E3 MVA T cell vector specific peptides was reduced in mice that received two vaccinations with Ad5+M compared to those that received two vaccinations of Ad5 or MVA respectively (Figure 4f). A similar pattern of anti-vector immunity amongst each group was observed with intracellular TNF-α production (data not shown). The summed anti-vector peptide specific immunity of combined average responses to each anti-vector MVA and Ad5 specific T cell specific peptide was reduced in mice that received the heterologous prime-boost regimen compared to mice that received the homologous Ad5+M prime-boost regimen (Figure 4f).

## Discussion

It is likely that future *M.tb* vaccination strategies will retain the use of BCG or a recombinant BCG strain. A successful improved tuberculosis vaccine regimen may require more than one booster vaccination following a BCG prime. Attenuated viral vectored vaccines offer a promising tool to boost immunity but the development of anti-vector immunity after vaccination may mean that repeated booster immunisations have to be suitably spaced in time, or that unique viral vectors may be required for sequential boosting. The logistics and costs to store and administer up to three different vaccines used in a tuberculosis vaccine regimen presents logistical and economical challenges.

Combining two viral vectors in one vaccine vial could have the advantage of reducing the costs of validating the potency of separate viral vectors if they were delivered as separate vaccines. Combining viral vectors into one vaccine may also reduce the logistical costs of storing and administering vaccines containing single viral vectors. We investigated whether mixing two different viral vectors that shared the Ag85A insert antigen in a single vaccine was a feasible method to reduce the logistical costs of a vaccination regimen.

In order for mixing different viral vectors to be worthwhile, two vaccinations with the mixture should be able to generate a greater level of immunity than two vaccinations with one viral vector and a comparable level as that observed with two vaccinations using different viral vectors. We hypothesised that mixing different viral vectors could achieve high levels of immunity by reducing anti-vector immunity against each vector in the mixture and boosting the recognition of the shared insert antigen Ag85A. We examined the level of anti-vector immunity by CD8+ T cells without measuring titres of vector specific antibodies. It would be possible to measure the level of anti-vector antibody titres in future experiments, however an observation of T cell specific anti-vector immunity does provide an insight to the extent that the T cell immune response is skewed away from recognition of Ag85A to recognition of the vector. This finding is important given that T cell mediated immunity is critical for protection against *M.tb* [5]–[7].

Our observations show a homologous prime-boost vaccine regimen using a mixture of two different viral vectors in a single vaccine does reduce anti-vector immunity to each viral vector compared to a prime-boost regimen using a single vector. However, the lowest level of anti-vector immunity was observed in the heterologous prime-boost regimen. These results correlated with the level of Ag85A-specific immunity induced by each regimen. The highest level of Ag85A-specific immunity and the lowest level of anti-vector immunity was observed in the heterologous prime-boost regimen.

Although mixing viral vectors clearly reduced anti-vector immunity and there was a trend for mixing to improve Ag85A-specific immunity, mixing did not consistently generate greater Ag85A-specific immunity compared to homologous doses of one viral vector. Furthermore, the level of Ag85A-specific immunity generated by a homologous prime-boost with the mixture was inferior to that generated using a heterologous prime-boost regimen using different viral vectors. This finding is supported by previous research to show that immune recognition of viral vector T cell epitopes detracts from the immune recognition of the insert antigen shared by the vaccine and pathogen [33]. Anti-vector specific antibody titres were not analysed here however future work to measure these responses could be important to further elucidate the benefit of different vaccination regimens incorporating viral vectors. Overall it appears that any logistical benefit to a vaccine regimen that might be obtained by delivering a vaccine containing a mixture of viral vectors would be outweighed by the disadvantages of reduced immunity to the shared insert antigen compared to delivering heterologous vaccinations of separate viral vectors. The lack of a clear benefit from mixing viral vectors to deliver two homologous boosts is also apparent after a BCG prime.

In contrast to results obtained in this study, other research has identified an enhancement of immunogenicity to malaria antigens using the Ad5+M regimen compared to homologous prime-boosting with single vectors, that correlated with an enhanced protection to liver stage malaria [34]. The difference between these studies may derive from a difference in immunodominance of the shared insert antigen compared to the anti-vector responses. A number of immune functions have been shown to be necessary but not sufficient for protection from *M.tb* [3], [4]. Ongoing research is attempting to disern which biomarkers can provide a signature of protection to *M.tb* that could be used to measured future vaccine efficacy in humans. It is possible that the benefit of mixing viral vectors to reduce anti-vector immunity, as shown in this study, will be shown to be beneficial in the context of future biomarks of protective *M.tb* specific immunity. The efficacy of new *M.tb* vaccines are routinely tested using the aerosol *M.tb* challenge model. However this model is known to lack sufficient sensitivity to measure if small alterations in immunogenicity between vaccine regimens translate into an improved efficacy. In the absence of clear benefit of homologous vaccinations using a mixture of viral vectors compared to a single vectored vaccine in this study, it is unlikely mixing would significantly improve vaccine efficacy in a model of *M.tb* challenge.

In summary, these results expand our understanding of how future vaccine strategies may be improved and are important for the field of tuberculosis and other T cell inducing vaccines. This study provides a platform to further analyse the immunogenicity of different vaccine regimens in order to establish how different vaccines interact to modify overall immunogenicity to antigens used in a particular vaccine regimen and establish methods to enhance immunogenicity but reduce the logistical and financial obstacles to delivering these regimen to the target populations with endemic disease.
